# In Vitro Construction of Scaffold-Free Bilayered Tissue-Engineered Skin Containing Capillary Networks

**DOI:** 10.1155/2013/561410

**Published:** 2013-03-27

**Authors:** Yuan Liu, Hailang Luo, Xinwen Wang, Akimichi Takemura, Yi Ru Fang, Yan Jin, Fumihiko Suwa

**Affiliations:** ^1^Department of Oral Histology and Pathology, School of Stomatology, The Fourth Military Medical University, Xi'an 710032, China; ^2^Xi'an Institute of Tissue Engineering & Regenerative Medicine, Xi'an 710032, China; ^3^Department of Periodontology & Oral Medicine, School of Stomatology, The Fourth Military Medical University, Xi'an 710032, China; ^4^Department of Anatomy, Osaka Dental University, 8-1 Kuzuhahanazono-cho, Hirakata-shi, Osaka 573-1121, Japan; ^5^Department of Dental Oriental Medicine, Osaka Dental University, 8-1 Kuzuhahanazono-cho, Hirakata-shi, Osaka 573-1121, Japan

## Abstract

Many types of skin substitutes have been constructed using exogenous materials. Angiogenesis is an important factor for tissue-engineered skin constructs. In this study, we constructed a scaffold-free bilayered tissue-engineered skin containing a capillary network. First, we cocultured dermal fibroblasts with dermal microvascular endothelial cells at a ratio of 2 : 1. A fibrous sheet was formed by the interactions between the fibroblasts and the endothelial cells, and capillary-like structures were observed after 20 days of coculture. Epithelial cells were then seeded on the fibrous sheet to assemble the bilayered tissue. HE staining showed that tissue-engineered skin exhibited a stratified epidermis after 7 days. Immunostaining showed that the epithelium promoted the formation of capillary-like structures. Transmission electron microscopy (TEM) analysis showed that the capillary-like structures were typical microblood vessels. ELISA demonstrated that vascularization was promoted by significant upregulation of vascularization associated growth factors due to interactions among the 3 types of cells in the bilayer, as compared to cocultures of fibroblast and endothelial cells and monocultures.

## 1. Introduction

Engineering skin substitutes hold promise for advanced treatment of acute and chronic skin wounds [1]. Skin tissue substitutes must readily adhere, have good physical and mechanical properties, and be nonantigenic [2]. Additionally, the substitutes should integrate into the host with minimal scarring and pain and facilitate angiogenesis [3]. Moreover, skin substitutes should restore functional anatomy and physiology after treatment and healing of the wound [4]. More recent skin substitutes combine epidermal and dermal layers by introducing fibroblasts and keratinocytes into an acellular matrix [5]. However, the engineering of more complex tissues consisting of large 3D structures remains a critical challenge because the penetration of oxygen, which is required for cell survival, is limited by diffusion to a distance of approximately 150 to 200 mm from the nearest blood vessel. Thus, the long-term survival and function of 3D tissues depend on the rapid development of new blood vessels to provide nutrients and oxygen to cells in the center of the tissue grafts. Sustained hypoxia or lack of nutrients leads to fibroblast dysfunction, decreased keratinocyte migration and proliferation, and tissue loss [6]. Hence, a major threat for the clinical use of dermal substitutes is insufficient vascularization leading to loosening, infection, or partial necrosis of the dermal substitute [7].

One classical approach to promoting vascularization is to decorate or supplement the scaffolds with proangiogenic factors such as VEGF, bFGF, and PDGF [8]. Different types of scaffolds containing these factors have been combined to enhance capillary formation in dermal wound healing models [9, 10]. However, these approaches often require repeated administration or control release systems [11]. Skin substitutes seeded with cells producing growth factor on a continuous basis provide an attractive alternative [12]. For example, seeding fibroblasts or keratinocytes results in faster vascularization due to growth factor secretion. Previous reports have shown that VEGF overexpression in keratinocytes augments wound vascularization [13]. In vitro prevascularization can result in faster integration with the host vascular network [14]. Most of these methods involve culturing endothelial cells on or within ECM substrates (e.g., collagen, fibrin, fibronectin, laminin, etc.) or other types of biomaterials to form capillary-like structures [15]. However, these methods have some drawbacks because the ECM substitutes contained exogenous materials such as bovine collagen, dead human allogenic dermis, or synthetic polymers [16]. Scaffold choice, immunogenicity, degradation rate, toxicity of degradation products, host inflammatory responses, fibrous tissue formation due to scaffold degradation, and mechanical mismatches with the surrounding tissue are key issues that may affect the long-term behavior of the engineered tissue constructs and directly interfere with their primary biological functions [17].

In recent years, a variety of techniques have been developed to engineer tissues composed only of cells and the matrix materials that they secrete without any exogenous scaffold materials. Such techniques are even being applied for tissues where scaffolds have shown success (e.g., skin, bone, and cartilage) [18]. Scaffold-free cell sheet-based constructs have been applied for tissue repair due to their high rates of nutrient diffusion, abundant deposition of ECM, and interactions with cell membrane proteins, including growth factors, ion channels, and cell-to-cell junction proteins. Ascorbic acid has been reported to induce telomerase activity, leading to upregulation of type I collagen, fibronectin, and integrin *β*1. Ascorbic acid-treated mesenchymal cells form sheets due to increased ECM production. Furthermore, Lee et al. constructed a dermal equivalent from fibroblasts treated with ascorbic acid without other exogenous materials [19]. Based on this paper, we cocultured 3 types of cells (keratinocytes, dermal fibroblasts, and dermal microvascular endothelial cells) to form scaffold-free skin equivalents containing capillary-like networks. The cocultured cells expressed high levels of vascularization-associated growth factors, including VEGF, bFGF, and PDGF, compared to cocultures of fibroblasts and endothelial cells and monocultures.

## 2. Materials and Methods

### 2.1. Media Preparation

- PBS (containing 10,000 U/mL penicillin and 10,000 *μ*g/mL streptomycin).
-   Dispase (1.2 U/mL).
-  Keratinocyte culture medium: K-SFM, Gibco.
-  Dermal fibroblast culture medium (DFM): Dulbecco's modified Eagles medium (DMEM) supplemented with 2% fetal bovine serum and 25 *μ*g/mL bovine pituitary gland extract (BPE).
-  Long-term culture medium (ECM) for endothelial cells: M199 supplemented with 20% fetal bovine serum, 25 *μ*g/mL endothelial cell growth supplements (ECGS), and 90 *μ*g/mL heparin.
-  Selective culture medium (ESM) for endothelial cells: DMEM supplemented with 10% fetal bovine serum and 150 *μ*g/mL G418.
-  Dermis substitute culture medium (DSM): DFM with 50 mg/mL ascorbic acid.

### 2.2. Cell Cultures

Three types of primary cells were established from neonatal foreskin. For human epidermal keratinocytes, the foreskin was rinsed in 70% ethanol for 1 min and then rinsed twice in PBS. The subcutaneous fat and deep dermis were excised, and the remaining tissue was cut into small pieces. The skin pieces were then incubated in 1.2 U/mL dispase (2.4 U/mL, Gibco BRL) at 4°C overnight. The tissue fragments were then transferred to a Petri dish, the epidermis and dermis were separated with fine forceps, and the epidermal sheets were transferred to a 15 mL centrifuge tube containing 0.25% trypsin and incubated at 37°C for 10 min. Gentle pipetting was used to disaggregate the epidermal sheets into single cell suspensions. The cells were then counted and seeded at 10^6^ cells per two 35 mm dishes. Cells were incubated in DFM containing 10% FBS for 1 h to facilitate attachment. The medium was then changed to KSFM. The cells were subcultured at 80% confluence. The remaining dermal parts were incubated in a preheated sterile collagenase solution (625 U/mL, Sigma) for 2 hours at 37°C. The dermal cells were then pipetted gently to dissociate them into a single cell suspension. Dermal fibroblasts were cultured in DFM. Endothelial cells were isolated using a Dynabeads CD31 Endothelial Cell Kit. The endothelial cells were cultured in ESM medium for 2 days to prevent fibroblasts contamination. The medium was then changed to ECM medium for long-term culture.

### 2.3. Flow Cytometry

The phenotypes of cultured epithelial cells, fibroblasts, and endothelial cells were identified by flow cytometry. The cultured cells were harvested using trypsin, washed twice with PBS, incubated with FITC-conjugated monoclonal antibodies (keratin 5, Factor VIII, and vimentin; all from BD Bioscience, USA), and analyzed on a FACScan. All experiments were performed at least in triplicate.

### 2.4. Coculture of Fibroblasts and Endothelial Cells

At passage 3, when fibroblasts and endothelial cells reached 70–80% confluence, the cells were digested with 0.25% trypsin-0.01% EDTA for 1 minute. The reaction was quenched with 10 mL of bovine serum. After 5 min of centrifugation, the fibroblasts and endothelium were resuspended in DSM at 1.0 × 10^5^ cells/mL. The fibroblasts and endothelial cells were cocultured on slides at ratio of 2 : 1 for more than 20 days.

### 2.5. Reconstruction of Scaffold-Free Bilayered Skin Containing Capillary-Like Structures

Dermal fibroblasts and endothelial cells were suspended in DSM at a concentration of 1.0 × 10^6^ cells/mL and seeded at a ratio of 2 : 1 for more than 4 weeks to produce a cell sheet. After the cell sheet was separated, four layers were superimposed and cultured for one additional week to produce a dermal equivalent. Epidermal keratinocytes were then seeded at a density of 1.0 × 10^5^ cells/mL on the dermal equivalent and cultured for one more week. Capillary-like structure localization was observed by HE staining.

### 2.6. Immunostaining

Cocultured cells were fixed for 30 minutes at room temperature in phosphate-buffered saline (PBS) containing Ca^2+^, Mg^2+^, and 4% paraformaldehyde. The fixed specimens were then transferred to 20% sucrose, frozen in optimum cutting temperature compound (Tissue Tek, Sakura, Japan), and immunostained for Factor VIII using standard protocols [20].

### 2.7. TEM

All protocols were according to Kunz-Shughart et al. report [21]. Bilayered engineering skin was fixed in 0.1 M cacodylate-buffered Karnovsky's solution (2.5% glutaraldehyde and 1% paraformaldehyde overnight at room temperature) and postfixed in 1% osmium tetroxide (2 h), pH 7.3, dehydrated in graded ethanol, and embedded in EmBed-812 epoxy resin (all reagents were obtained from Science Services, Munich, Germany; automated LYNX tissue processor, Leica, Germany). After 48h heat polymerization at 60°C, semithin (0.8 *μ*m) sections were cut, stained with toluidine blue/fuchsin, and after light microscopic spheroid selection, the epon block was trimmed for ultrathin sectioning. Ultrathin (80 nm) sections were prepared with a diamond knife on a Reichert Ultracut-S ultramicrotome and double contrasted with aqueous 2% uranyl acetate and lead citrate solutions for 10 min each. The sections were examined using a LEO912AB electron microscope operating at 80 kV.

### 2.8. ELISA

To compare the expression of vascularization associated growth factor under different culture conditions ((a) epithelial cell, fibroblast, and endothelial cell coculture; (b) fibroblast and endothelial cell coculture; (c) fibroblast monocultures; (d) epithelial cell monocultures; and (e) endothelial cellmonocultures), VEGF, bFGF, and PDGF expressions were measured using enzyme-linked immune sorbent assay (ELISA) kits (R&D Systems, USA). ELISA plates were coated with monoclonal capture antibodies and blocked with bovine serum albumin (1 w/v%) and sucrose (5 w/v%) for 1 h. Bound VEGF, bFGF, and PDGF were detected using biotin-conjugated anti-human VEGF, bFGF, and PDGF monoclonal antibodies. Streptavidin-conjugated horseradish peroxidase was added to the plates, and an enzyme substrate (tetramethylbenzidine and peroxide) was added for 20min. The reaction was quenched by adding an acidic solution, and absorbance was read at 450 nm using a PowerWave X340 plate reader (BioTEK Instruments, Inc., USA). Experiments were performed using five replicates of each sample.

### 2.9. Statistical Analysis

Data are expressed as mean SD. Analysis was performed using the Statistical Program for Social Science (SPSS) 13.0 for Windows. Analysis of variance followed by Student's *t*-test was used to determine the significant differences among the groups, and *P* values less than 0.05 were considered significant.

## 3. Results

### 3.1. Cell Growth and Identification

**Figure 1:**
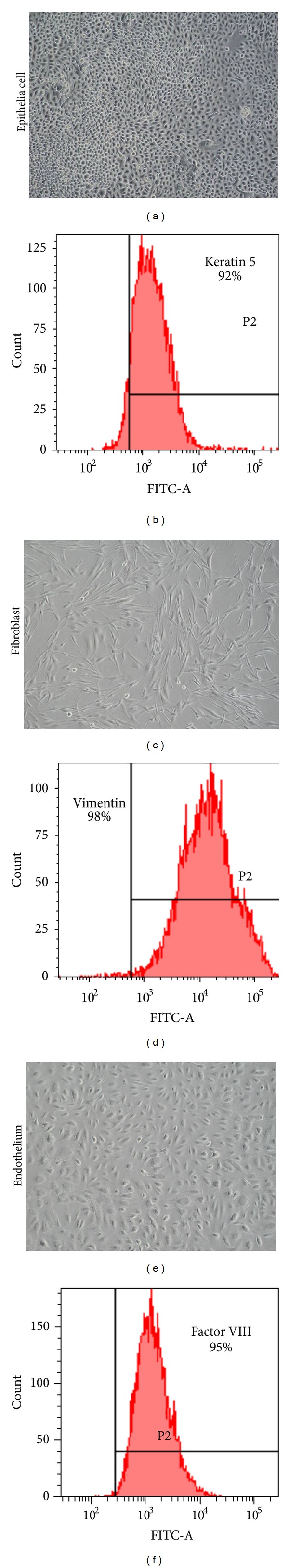
(a) Morphological observation of epithelium culture on Petri dish by light microscope at passage 3. (b) Keratin 5 flow cytometry analysis; the positive rate reached to 92%. (c) Morphological observation of fibroblast culture on Petri dish by light microscope at passage 3. (d) Vimentin flow cytometry analysis; the positive rate reached to 98%. (e) Morphological observation of endothelium culture on Petri Dish by light microscope at passage 3. (f) Factor VIII flow cytometry analysis; the positive rate reached to 95%.

Epithelial cells isolated from neonatal foreskin displayed typical cobblestone morphology until at least passage 10 (Figure 1(a)). At passage 3, 92% of epithelial cells were keratin 5 positive as determined by flow cytometry (Figure 1(b)). Dermal fibroblasts displayed bipolar spindle morphology until at least passage 20 (Figure 1(c)). At passage 3, 98% of dermal cells were vimentin-positive by flow cytometry (Figure 1(d)).

Endothelial cells have a characteristic appearance that distinguishes them from other cell types. At low density, primary endothelial cells are elongated. However, when 70–80% confluence is reached (or after subculture), endothelial cells display a characteristic cobblestone morphology for up to 15 passages (Figure 1(e)). Primary endothelial cultures were subcultured when cells reached 70–80% confluence. After 2 days in culture, fibroblast contamination was observed in some areas. However, after culture in ESM for another 3 days, fibroblasts contamination was eliminated. Endothelia cells can be maintained in ECM for a long period of time without obvious changes in cell morphology or proliferation. Up to 95% of endothelial cells were Factor VIII-positive by flow cytometry (Figure 1(f)).

### 3.2. Cocultures of Endothelial Cells and Fibroblasts

**Figure 2:**
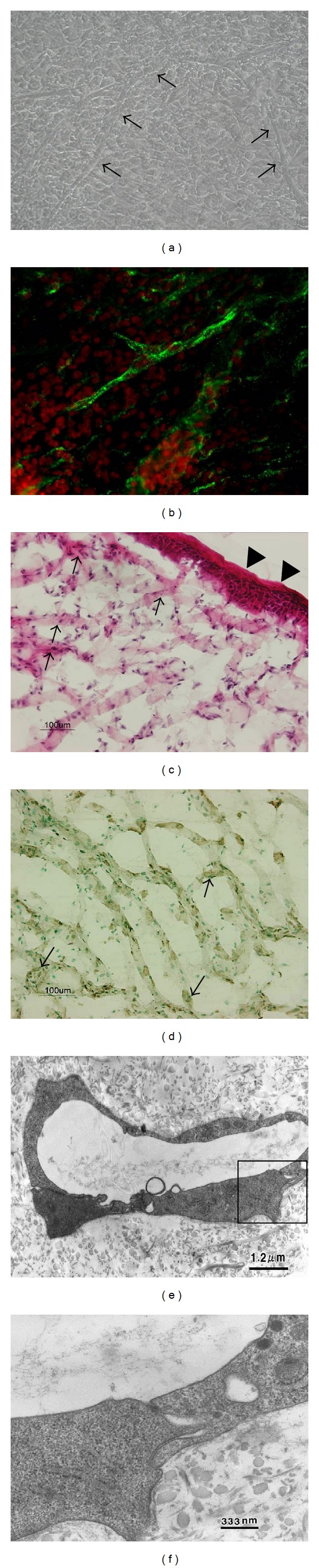
(a) The morphological observation of coculture of fibroblast and endothelium after 20 days. Abundant matrix secretion and lumen-like structures could be observed by light microscopy. Arrows point to the lumen-like structure. (b) Factor VIII immunofluorescence showed that the lumen-like structures were formed by endothelial cells and distributed throughout the construct. Green was the Factor VIII positive staining, and red was the cell nuclei. (c) HE staining of bilayered tissue-engineered skin. HE staining showed that the skin exhibited a stratified epidermis composed of a cuboidal basal layer, suprabasal layers, a granular layer expressing filaggrin and transglutaminase, and a stratum corneum. In dermis, a large number of cells gathered in the collagen fiber. Triangles point to the epidermis, and arrows point to the dermis. (d) Factor VIII immunostaining of bilayered tissue-engineered skin. Dark brown stain was positive for factor VIII. Results showed a large number of factor VIII expressed endothelium distributed in dermis forming lumen-like structure. Arrows point to the lumen-like structure. ((e) and (f)) TEM observation of ultrastructure of capillary in bilayered tissue-engineered skin. The morphology showed that capillary had a typical micro-blood vessel structure.

When endothelial cells and fibroblasts were cocultured, the fibroblasts proliferated and synthesized new matrix materials. At 20 days, abundant matrix secretion was observed. Lumen-like structures were also observed by light microscopy (Figure 2(a)). Moreover, factor VIII immunofluorescence showed that the lumen-like structures were formed by endothelial cells and distributed throughout the construct. This result suggests that dermal fibroblast specific matrix proteins support endothelia cell migration and the formation of a capillary-like tubular structure (Figure 2(b)).

### 3.3. Reconstruction of the Skin Equivalent Containing Capillary-Like Structures

After 5 weeks of fibroblast-endothelium coculture, epithelial cells were seeded on the constructs to form bilayered skin. One week after-seeding, the skin was harvested from the culture dish. The skin equivalents were approximately 1 mm. HE staining showed that the skin exhibited a stratified epidermis composed of a cuboidal basal layer, suprabasal layers, a granular layer expressing filaggrin and transglutaminase, and a stratum corneum (Figure 2(c)). Moreover, Factor VIII immunostaining revealed that interactions between epithelial cells and fibroblast enhanced capillary-like network formation, as compared to cocultures of endothelial cells and fibroblasts (Figure 2(d)). To verify that the capillary-like network was composed of microblood vessels, TEM was used to visualize the ultrastructure of lumens in the tissue-engineered skin. The endothelium component of the dermis formed typical micro-blood vessel structures (Figures 2(e) and 2(f)).

### 3.4. Epithelial Cells Promote New Blood Vessel Formation in Tissue-Engineered Skin

**Figure 3:**
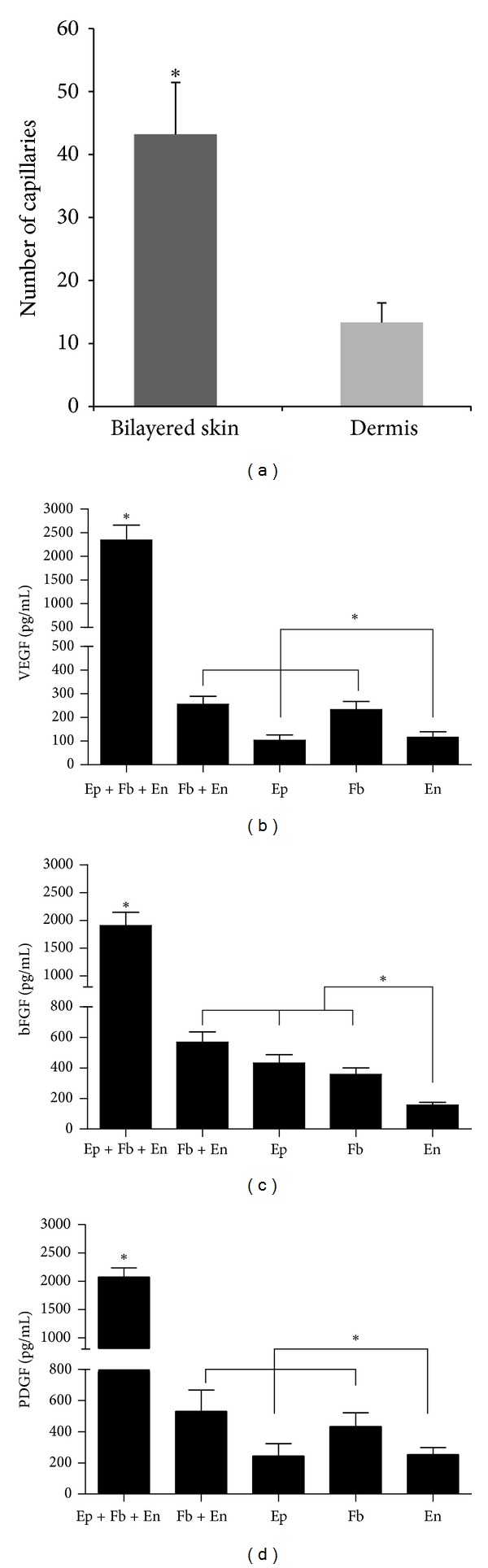
(a) The comparison of capillary network between bilayered engineering skin and dermis by image analysis. (b) The comparison of VEGF expression during different culture conditions by ELISA analysis. (c) The comparison of bFGF expression during different culture conditions by ELISA analysis. (d) The comparison of PDGF expression during different culture conditions by ELISA analysis.

Capillaries were nearly 3-fold more abundant in bilayered skin than in dermis (Figure 3(a)). Interactions between epithelial cells and fibroblasts regulate growth factor expression [22]; therefore, ELISA was used to compare the expression of vascularization-associated growth factors across the 5 groups. Expressions of VEGF, bFGF, and PDGF were upregulated 6-, 3-, and 4-fold, respectively, in epithelial cell, fibroblast, and endothelial cell cocultures compare to fibroblast and endothelial cell co-cultures (Figures 3(b), 3(c), and 3(d)). VEGF expression was not significantly different between fibroblast monocultures and co-cultures of fibroblast and endothelial cells; however, VEGF expression was significantly higher in fibroblast monocultures compared to epithelial cell and endothelial cell monocultures (Figure 3(b)). PDGF expression followed a similar trend to VEGF expression (Figure 3(d)). bFGF expression was significantly lower in endothelial cell monocultures compared to the other groups, although the differences among the remaining 3 groups were not significant (Figure 3(c)).

## 4. Discussion

Tissue engineering seeks to replace damaged, injured, or missing tissues with biologically compatible substitutes [23]. Tissue-engineering substitutes offer many advantages over traditional therapies [24]. However, the engineering of more complex tissues consisting of large 3D structures is challenging due to limitations in vascularization and angiogenesis. Oxygen and nutrients required for cell survival become limiting at approximately 150 to 200 mm from blood vessel; therefore, cells far from the wound surface die from hypoxia or lack of nutrients [25]. Accordingly, tissue-engineering research has focused on understanding the formation of new blood vessels. Skin substitutes formed in vitro with vascular networks inosculate faster with the host vascular network [26]. This study describes an approach to construct scaffold-free bilayered skin containing capillary networks by epithelial cells, fibroblasts, and endothelial cells. In our system, epithelial cells and cocultured fibroblasts secrete specific matrix components that stimulate endothelial cells to form capillary networks in the dermis. We found that cross-talk among these 3 cell types upregulates the expression of vascularization associated growth factors, including VEGF, bFGF, and PDGF.

Endothelial cell co-cultures represent a starting point for vascularization [27]. Endothelial cell spheroids produce capillary-like sprouts, especially in the presence of fibroblasts. Fibroblasts modulate endothelial cell network formation, suggesting a critical need for complex mixed spheroid cocultures to adequately mimic in vivo angiogenesis [21]. Fibroblasts are also a rich source of growth factors for self-stimulation and for activation of other cell types. Activated fibroblasts produce angiogenic growth factors, such as VEGF, bFGF, and PDGF [28]. Seeding only fibroblast in a scaffold can thus promote vascularization due to growth factor secretion [12]. We obtained similar results when cocultured dermal fibroblasts caused hDMVECs to migrate and form capillary-like tubular structures in the fibroblast-derived matrix. Furthermore, capillary formation was enhanced by seeding epithelial cells due to the upregulation of vascularization associated growth factors. These results were not improved by increasing the number of cells, suggesting that cross-talk occurred among epithelial cells, fibroblasts, and endothelial cells. Our ELISA results demonstrate that VEGF, bFGF, and PDGF are also upregulated in epithelial cell cocultures compared to cultures without epithelial cells containing the same number of cells. The interactions between epithelial cells and fibroblasts are intimately involved in a number of functions within the skin, including tissue differentiation during skin development, responses to and regulation of local inflammatory factors, and repair of damaged tissue [29]. Thus, these interactions mimic skin development in vivo, enhancing the formation of 3D vessel-like structures.

Extracellular matrix components also play important roles in blood vessel formation [30–32]. Endothelial cells require adhesion molecules and growth factors to promote their growth and tubular organization. In vitro angiogenesis models usually combine endothelial cells with various purified extracellular matrix components, such as collagen [33], fibrin [34], and fibrinogen [35], or multimolecular matrices such as Matrigel [36]. Thus, exogenous ECM is usually applied to form capillary-like structures. However, these exogenous biomaterials may impart unforeseeable cell–biomaterial interactions, uneven degradation, inflammatory responses, and infection, or may limit cell adhesion [37]. To avoid these disadvantages, we prepared bilayered tissue-engineered skin without any exogenous biomaterials. The matrix components of the dermis were secreted by fibroblasts. Fibroblast-derived matrix proteins, such as collagen 1, PCOLCE, SPARC, IGFBP7, and *β*ig-h3, induce endothelial cell sprouting and are necessary for lumen formation [38]. In addition, dermis-specific matrix components secreted by fibroblasts deposit numerous growth factors and adhesion molecules that can promote blood vessel formation and skin repair [39, 40]. Therefore, tissue-engineered skin has a strong capacity for forming capillary networks.

## 5. Conclusion

We described an approach to construct scaffold-free bilayered skin containing capillary networks from epithelial cells, fibroblasts, and endothelial cells. We first prepared a dermal sheet from fibroblasts and endothelial cells. After 20 days in culture, epithelial cells were seeded on the scaffold-free dermis to form bilayered skin. Capillaries were 3-fold more abundant in bilayered skin than in dermis. The capillary-like networks contained typical micro-blood vessels. ELISA showed that interactions among the 3 cell types upregulated vascularization-associated growth factor expression compared to cocultures of fibroblast and endothelial cells and monocultures.
